# Supplementary material for: Broken translational symmetry at edges of high-temperature superconductors
Source: Nat Commun. 2018 Jun 6;9:2190. doi: 10.1038/s41467-018-04531-y (PMC5989275; doi:10.1038/s41467-018-04531-y)
Supplement: Supplementary file 1 — Supplementary Information [file 41467_2018_4531_MOESM1_ESM.pdf]

Broken translational symmetry at edges of high-temperature superconductors

Holmvall *et al.*

Supplementary Figures

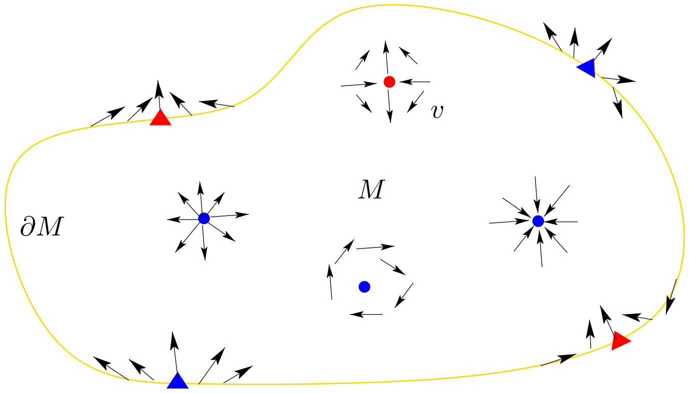

Supplementary Figure 1: **Schematic representations of typical critical points.** The critical points of the $\hat{\mathbf{p}}_{\mathrm{s}}(\mathbf{R})$ vector field inside the manifold are denoted by blue $(+1)$ and red (-1) dots. For the critical points of the tangent field on the boundary of the manifold we use blue $(+1)$ and red (-1) triangles. The triangle tip points inside or outside depending on the direction of the normal field at the critical point of the tangent field. The inside-pointing indices come with $(+1/2)$ weight, whereas outside-pointing ones come with $(-1/2)$ weight in the general expression Eq. (3) of the main text.

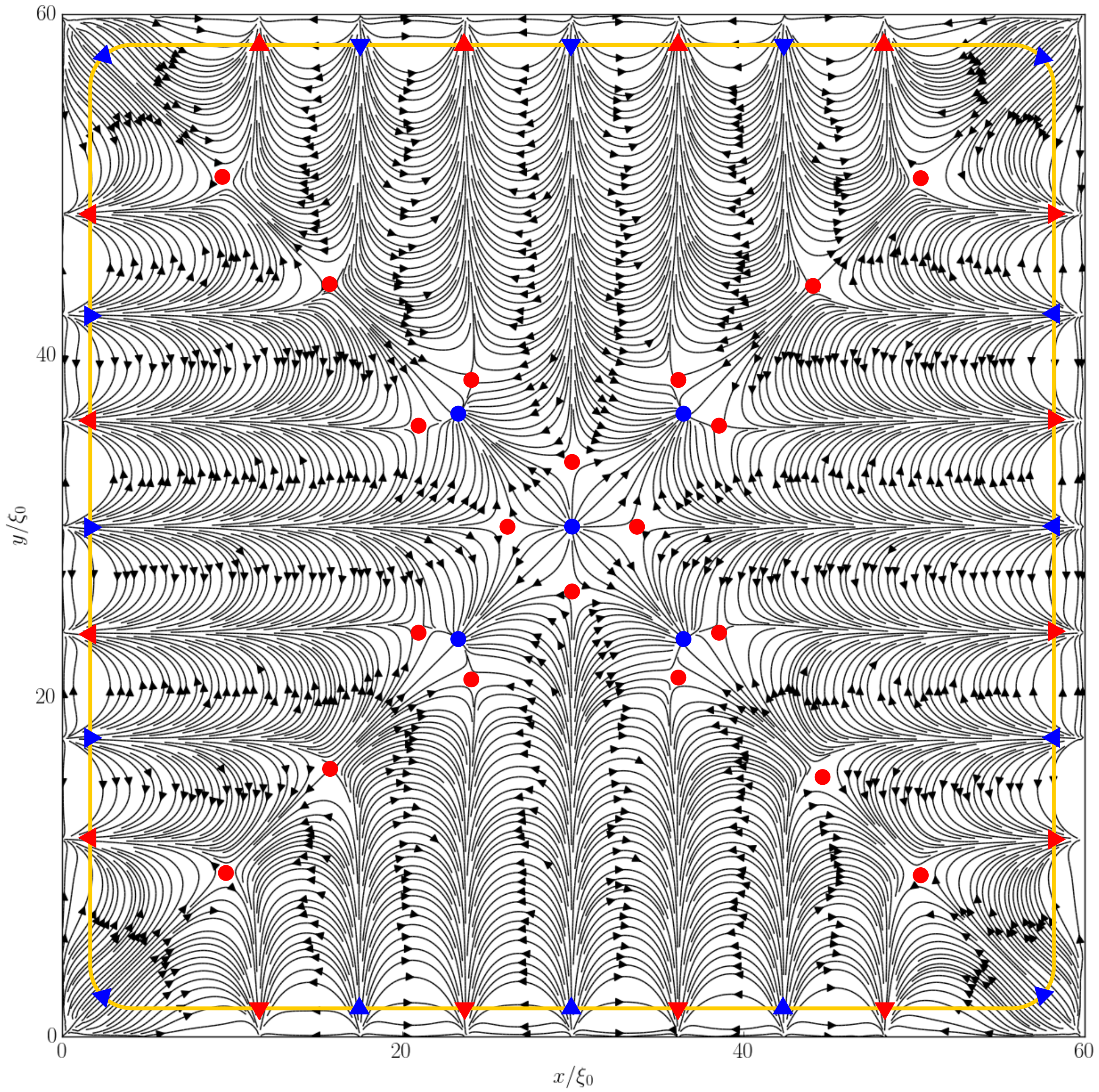

Supplementary Figure 2: **Critical points in the superflow vector field.** The indices of the critical points inside the volume are related to the critical points on the edge of the sample through the generalized Poincaré-Hopf theorem. We define a manifold boundary that circles the sample just inside the physical edge. It passes close enough to the sources and sinks of the superflow at the edge of the sample so that the critical points of the tangent field are determined by these. The 16 blue triangles indicate boundary critical points with index +1 and $\mathbf{p}_s$ field pointing inwards, while the 16 red triangles mark boundary critical points with index −1 and field pointing outwards. The red dots in the interior mark 20 saddle points (index $I = -1$ each) while the 5 blue dots mark sources or sinks (index $I = +1$ each).

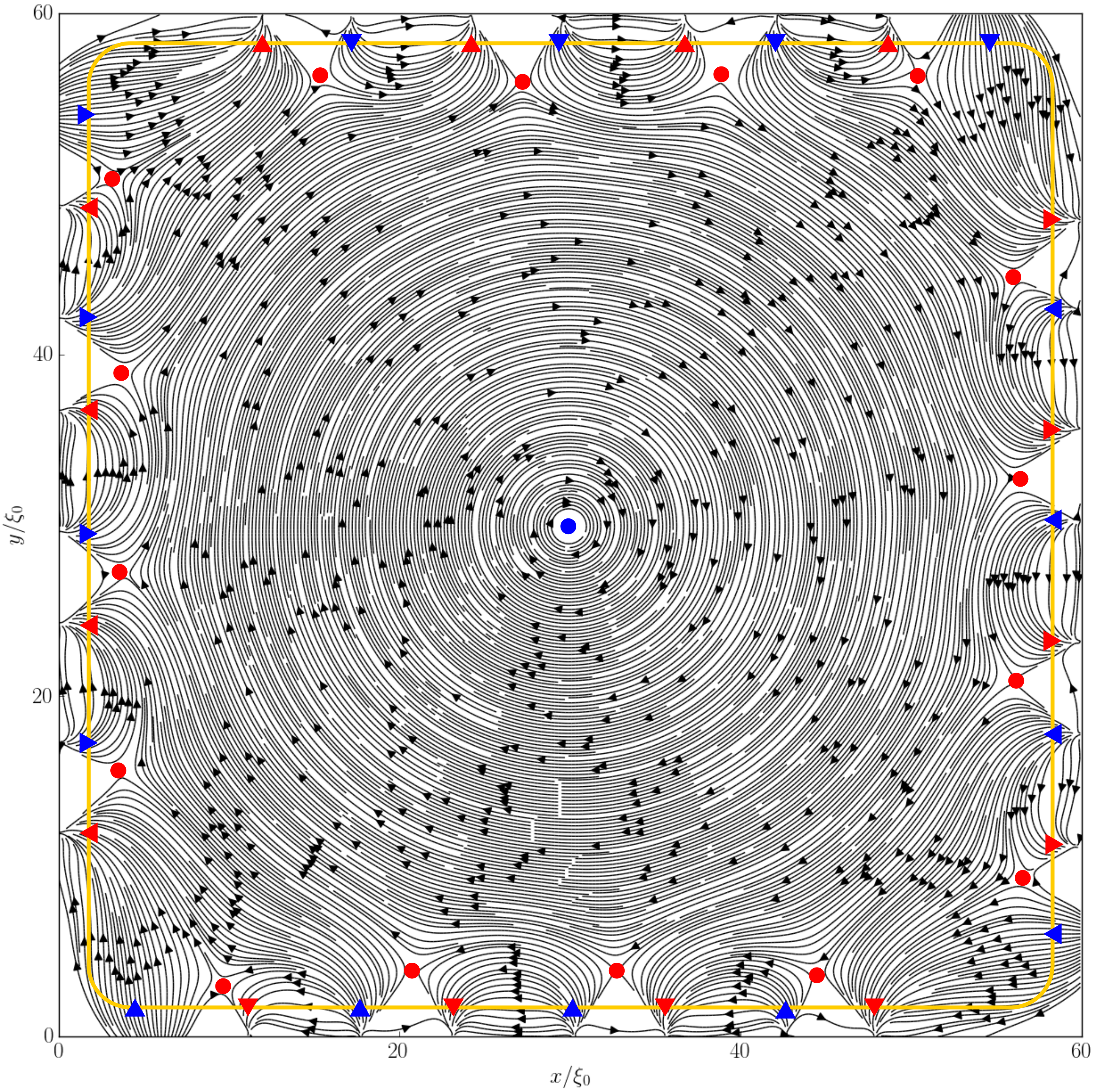

Supplementary Figure 3: **Critical points in uniform external magnetic field.** Critical points for a square grain with a uniform external magnetic field $B_{\text{ext}} = 0.5B_{\text{g1}}$, where $B_{\text{g1}} = \Phi_0/\mathcal{A}$ with flux quantum Φ_0 over the grain area $\mathcal{A} = 60\xi_0 \times 60\xi_0$. The Poincaré indices inside the boundary sum to $1 - 16 = -15$ due to 1 center (blue dot) and the 16 saddle points (red dots), while it sums to $[16 \times (+1) - 16 \times (-1)]/2 = +16$ on the boundary. The total index thus sums to the expected Euler characteristics for a square $16 - 15 = 1 = \chi(\text{square})$. Note that the absence of certain field lines is a technical shortcoming of the software used for plotting. See the corresponding quiver plot in Supplementary Figure **4** for greater detail.

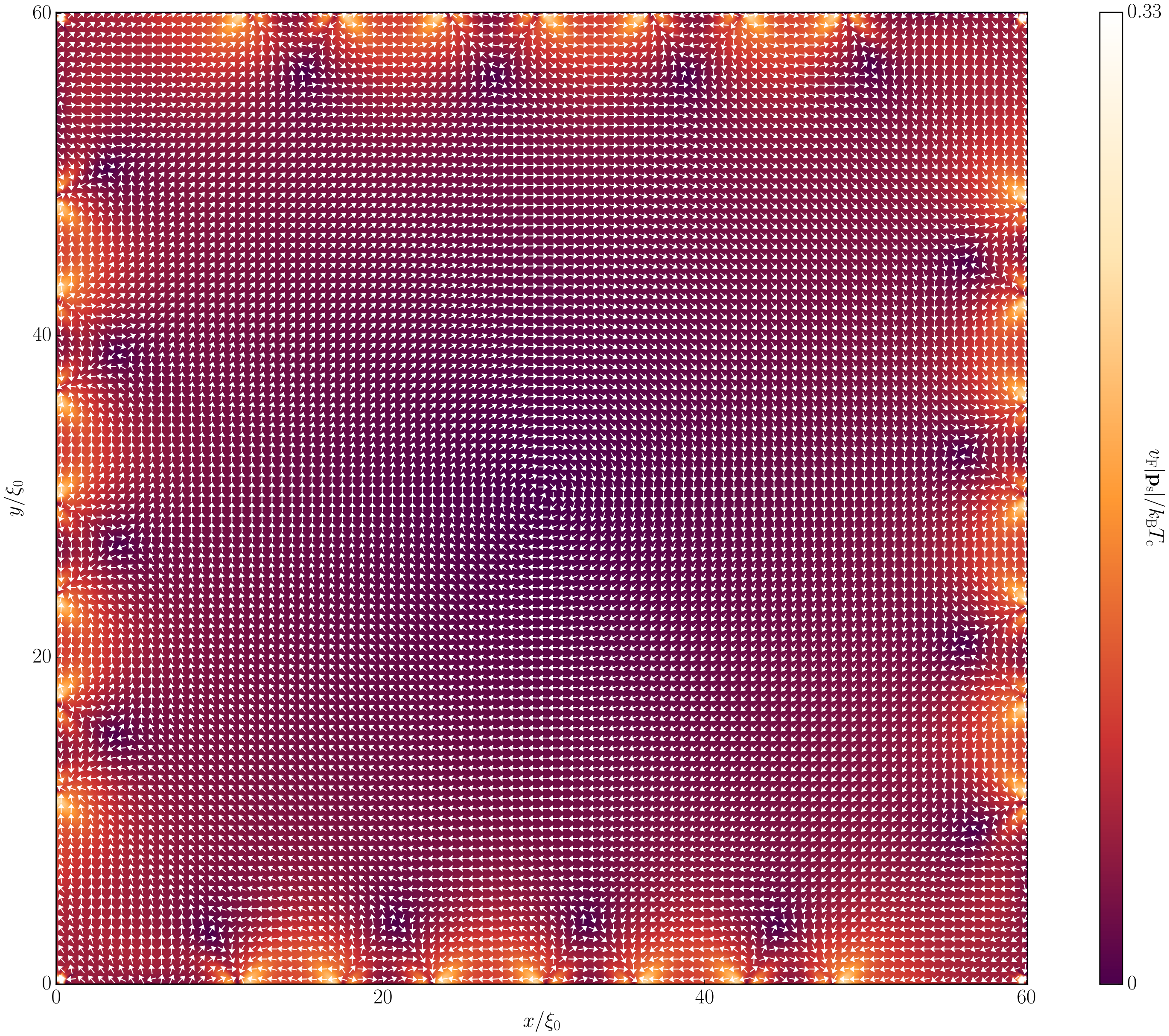

Supplementary Figure 4: **Superflow field in uniform external magnetic field.** The magnitude and direction of $\mathbf{p}_s(\mathbf{R})$ from Supplementary Figure **3**.

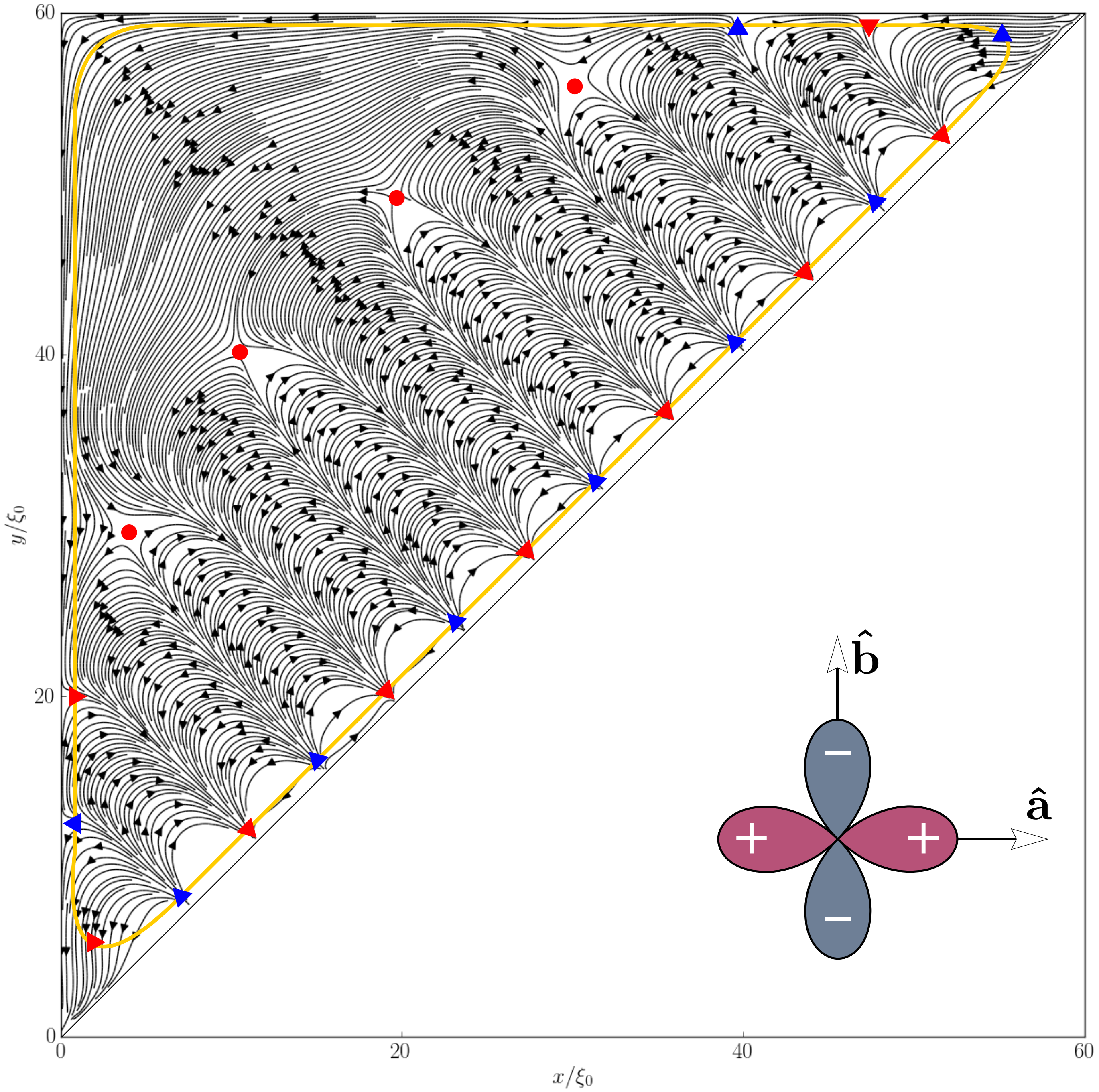

Supplementary Figure 5: **Critical points for a triangular grain.** For the triangle with the crystal ab-axes oriented as indicated in the drawing, we have a single pair breaking edge. The Poincaré indices inside the boundary sum to $4 \times (-1) = -4$ due to the 4 saddle points (red dots), while it sums to $[7 \times (+1) + 2 \times (-1) - 2 \times (+1) - 7 \times (-1)]/2 = 5$ on the boundary. The total index thus sums to the expected Euler characteristics for the triangle $5 - 4 = 1 = \chi(\text{triangle})$.

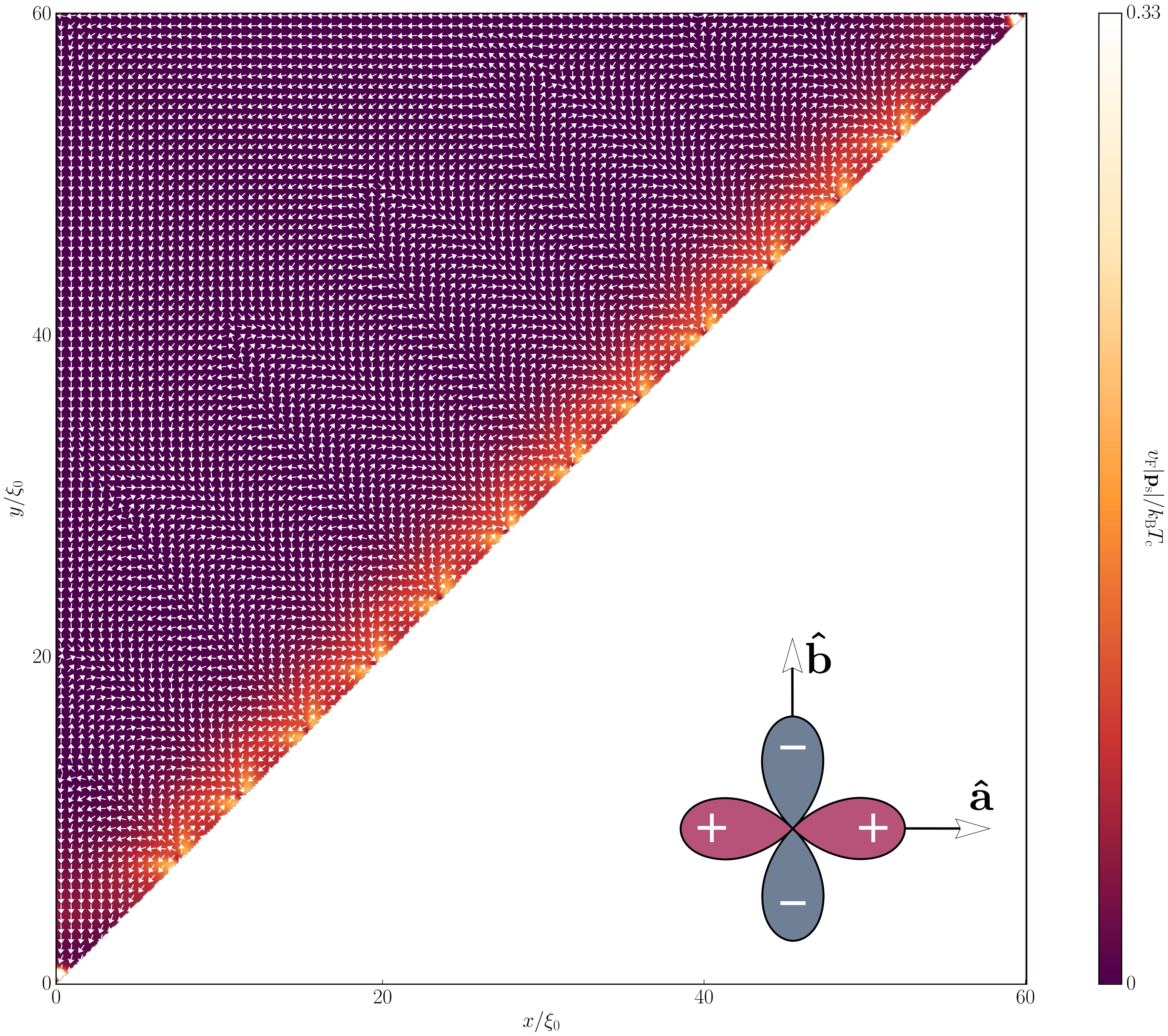

Supplementary Figure 6: **Superflow field for a triangular grain.** The magnitude and direction of $\mathbf{p}_s(\mathbf{R})$ from Supplementary Figure **5**.

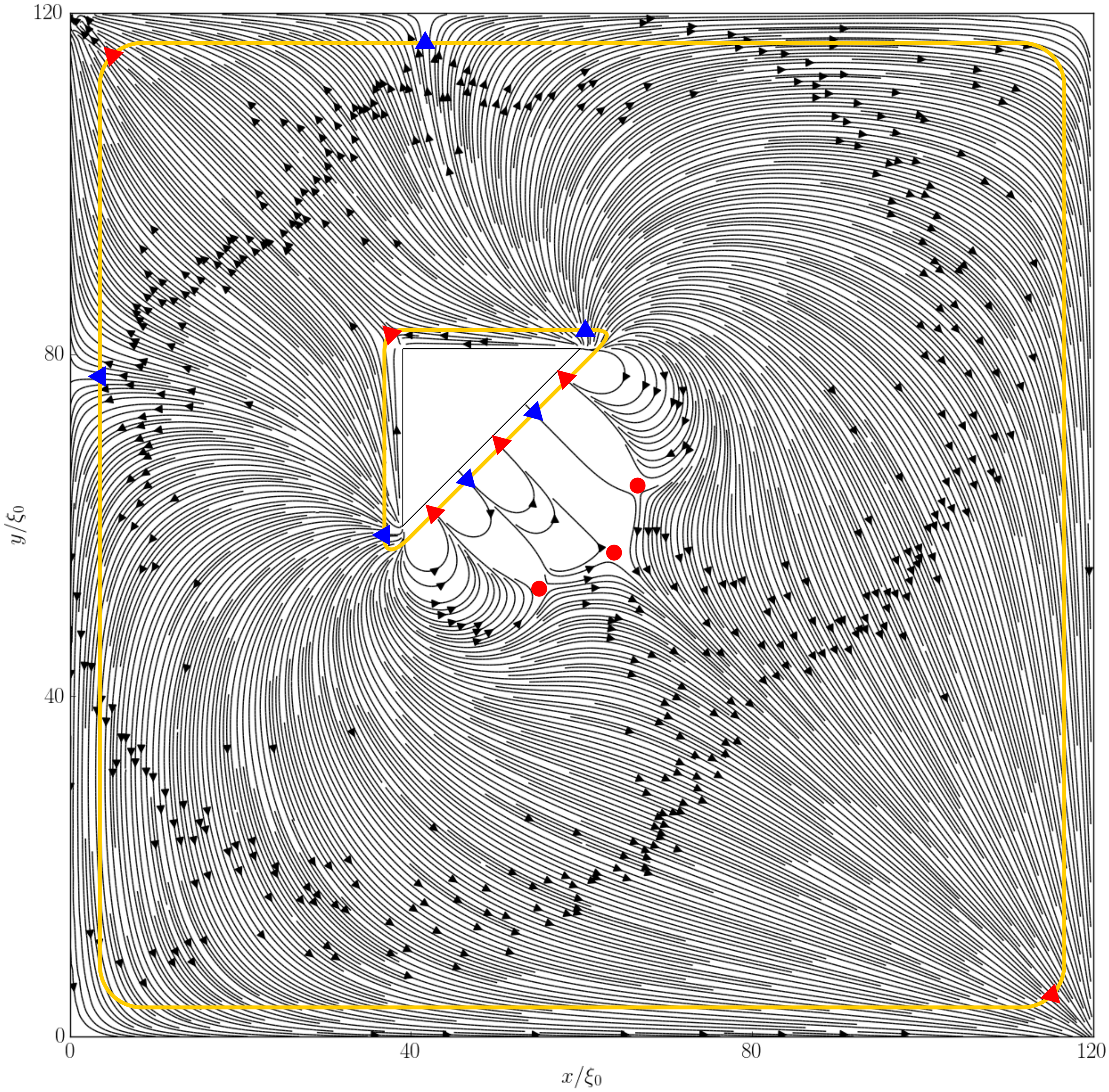

Supplementary Figure 7: **Critical points for a square grain with a hole.** The Poincaré indices between the outer and inner boundaries sum to $3 \times (-1) = -3$ due to the 3 saddle points (red dots), while it sums to $[-2 \times (+1) - 2 \times (-1)]/2 = 0$ on the outer boundary and $[4 \times (+1) + 1 \times (-1) - 3 \times (-1)]/2 = 3$ on the inner boundary. The total index thus sums to the expected Euler characteristics for a square with one hole $3 - 3 + 0 = 0 = \chi(\text{square} + 1\ \text{hole})$.

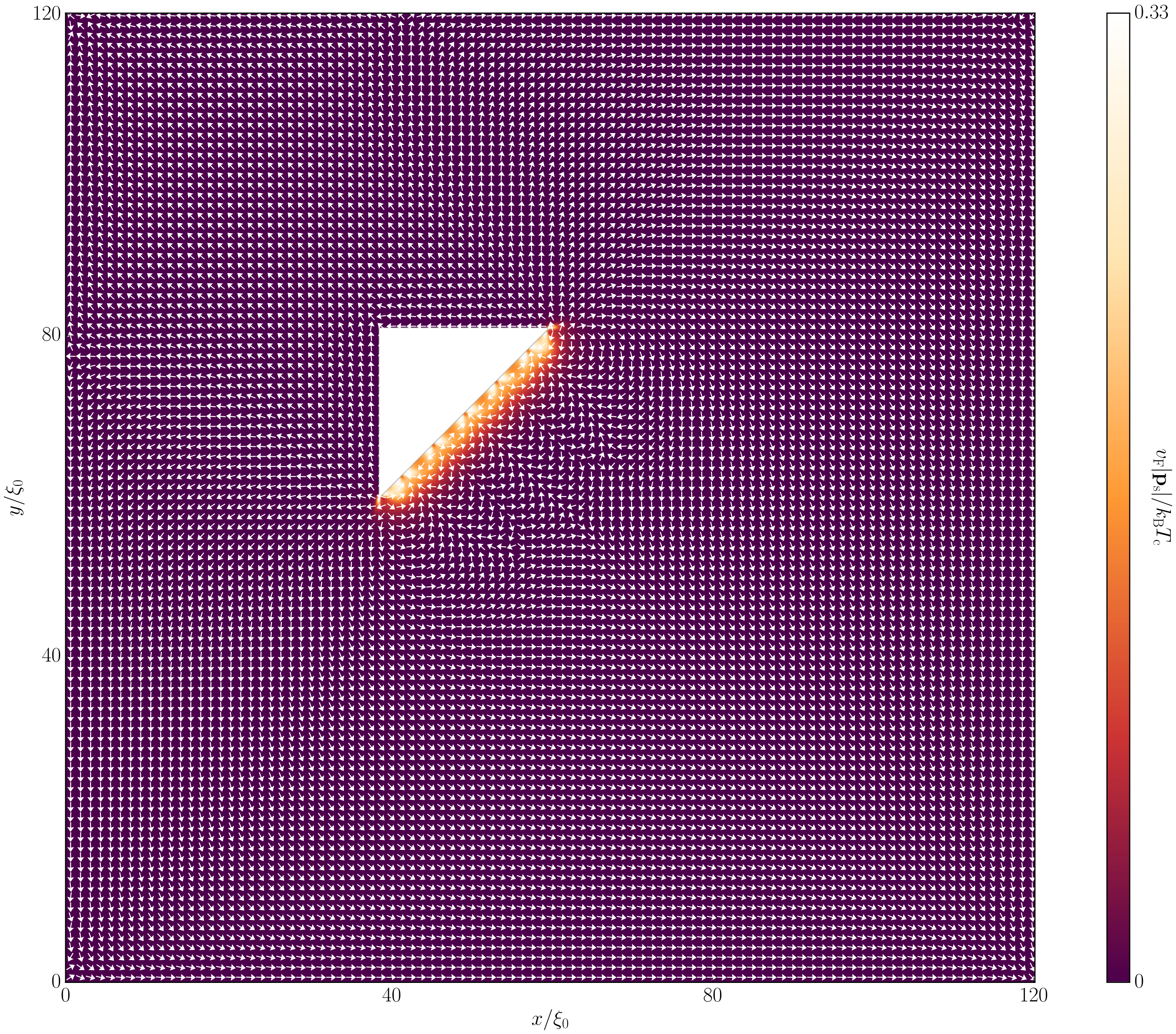

Supplementary Figure 8: **Superflow field for a square grain with a hole.** The magnitude and direction of $\mathbf{p}_s(\mathbf{R})$ from Supplementary Figure **7**.

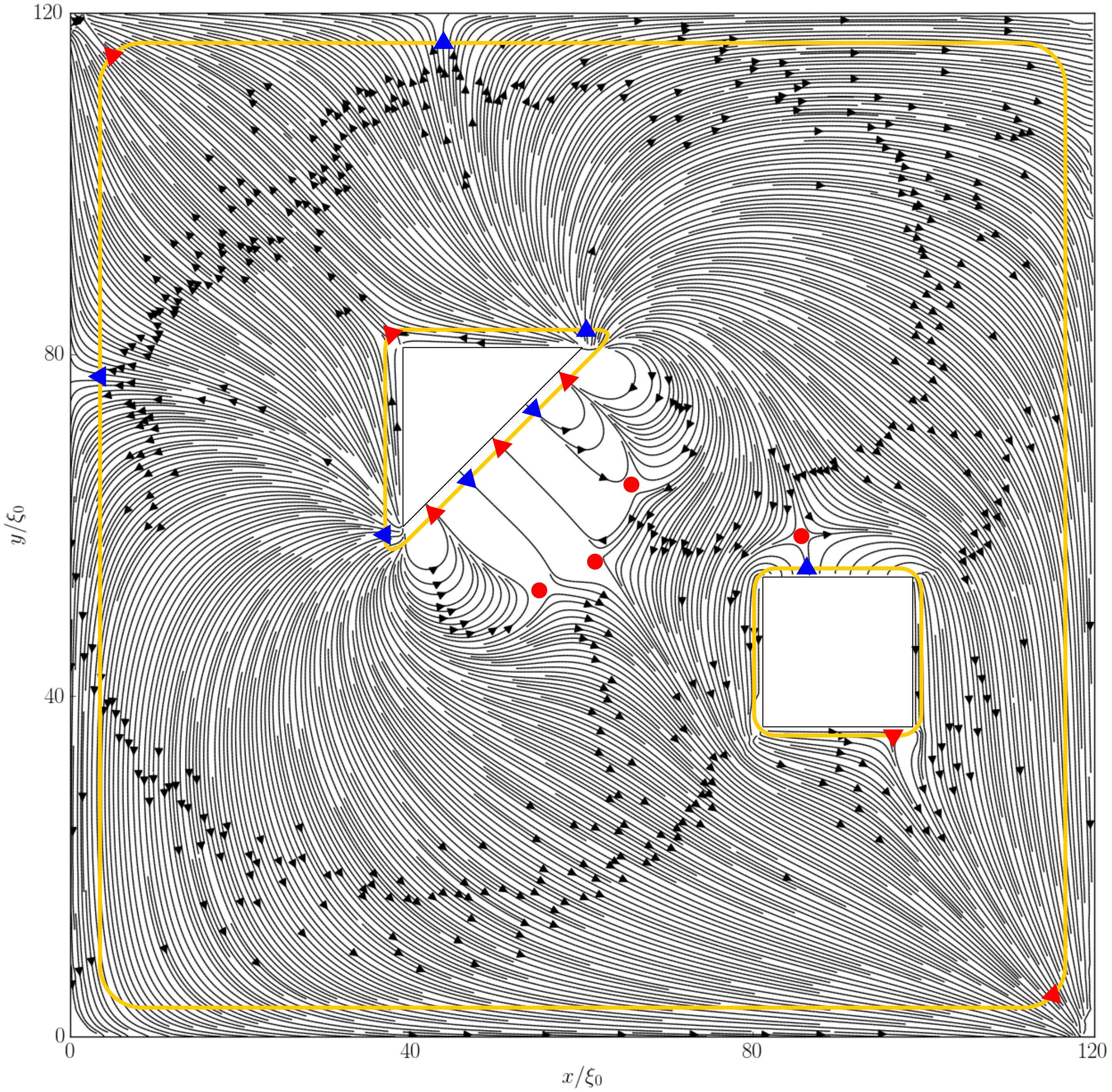

Supplementary Figure 9: **Critical points for a square grain with two holes.** The Poincaré indices between the outer and inner boundaries sum to $4 \times (-1) = -4$ due to the 4 saddle points (red dots), while it sums to $[-2 \times (+1) - 2 \times (-1)]/2 = 0$ on the outer boundary, $[4 \times (+1) + 1 \times (-1) - 3 \times (-1)]/2 = 3$ on the inner triangular boundary, and $(1 \times (+1) + 1 \times (-1))/2 = 0$ on the inner square boundary. The total index thus sums to the expected Euler characteristics for a square with two holes $3 - 4 + 0 + 0 = -1 = \chi(\text{square} + 2 \text{ holes})$.

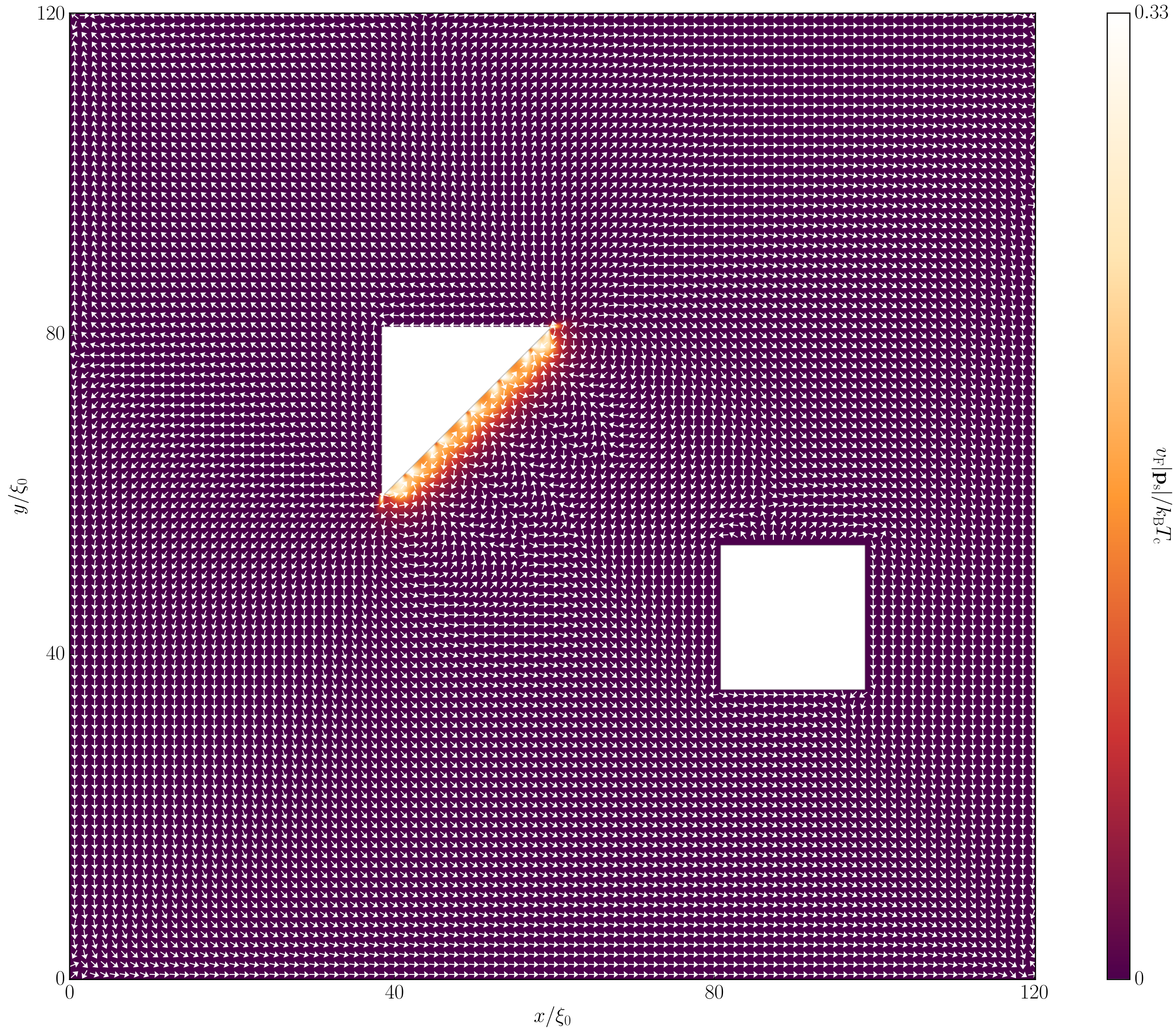

Supplementary Figure 10: **Superflow field for a square grain with two holes.** The magnitude and direction of $\mathbf{p}_s(\mathbf{R})$ from Supplementary Figure **9**.

Supplementary Notes

Supplementary Note 1. The Poincaré-Hopf theorem at work

We apply the generalized Poincaré-Hopf theorem to the vector field $\mathbf{p}_\mathrm{s}(\mathbf{R})$ of the new phase described in the main text, for various geometries and manifolds. In Supplementary Figure **2** we present the same vector field $\mathbf{p}_\mathrm{s}(\mathbf{R})$ as in Fig. 1 in the main text, but this time as a streamline plot. The locations of internal, two-dimensional, critical points are marked by colored dots, where the Poincaré index of saddle points is $I = -1$ (red dots), while the index for sinks, sources and centers is $I = +1$ (blue dots), see Supplementary Figure **1**. In Supplementary Figure **2**, these internal indices sum up to -15. In the formulation of the theorem, we are instructed to draw a boundary a small distance away from the sample edge, see the orange line. The one-dimensional critical points on this boundary, where the tangent vector of the field vanishes, are marked with colored triangles. These critical points are sinks (red triangles) and sources (blue triangles) with Poincaré indices $I = -1$ and $I = +1$, respectively. The direction of the triangle denotes the direction of the perpendicular vector, and hence if the index of the critical point is to be added ($\mathbf{p}_\mathrm{s}$ flowing in) or subtracted ($\mathbf{p}_\mathrm{s}$ flowing out). We count $16 \times (+1)$ critical points on the boundary with $\mathbf{p}_\mathrm{s}$ flowing in, and $16 \times (-1)$ points with $\mathbf{p}_\mathrm{s}$ flowing out. The theorem Eq. (3) in the main text gives

$$-15 + \frac{1}{2}\Big[16 - (-16)\Big] = 1 = \chi(\text{square}),$$

where the Euler characteristic of a square is $\chi(\text{square}) = 1$. Supplementary Figures **3** and **4** show that the theorem holds in the presence of an external magnetic field, while Supplementary Figures **5** and **6** show that it holds for other manifolds of the same kind, e.g. a triangle with a single pair-breaking edge, $\chi(\text{triangle}) = 1$.

To demonstrate that the theorem holds for other kinds of manifolds, superconducting grains with one and two holes are studied in a similar manner in Supplementary Figures **7**–**10**. For every hole that is added to the geometry, an additional boundary has to be drawn close around each additional hole, and the theorem states that the total Euler characteristic is reduced by one for each hole, i.e. $\chi(\text{square} + N \text{ holes}) = 1 - N$. Note that in contrast to the grains in the main paper and in Supplementary Figures **2**–**4**, where the crystal *ab*-axes are rotated 45° with respect to the outer edges of the system, the crystal *ab*-axes are aligned with the outer edges in Supplementary Figures **5**–**10**.

One of the consequences of the Poincaré-Hopf theorem is that the edges of a superconductor will host an even number of special points of the superflow field. This follows from the fact that all the indices are ± 1 and the Euler characteristic is an integer too. How these critical points of the superflow field distribute themselves on the edge, depends on the shape of the sample and geometrical symmetries.
